# Testing the Limit: Evaluating Drinking Water Arsenic Regulatory Levels Based on Adverse Pregnancy Outcomes in Bangladesh

**DOI:** 10.3390/toxics10100600

**Published:** 2022-10-11

**Authors:** Faye V. Andrews, Adam Branscum, Perry Hystad, Ellen Smit, Sakila Afroz, Mostofa Golam, Omar Sharif, Mohammad Rahman, Quazi Quamruzzaman, David C. Christiani, Molly L. Kile

**Affiliations:** 1 School of Biological and Population Health Sciences, College of Public Health and Human Sciences, Oregon State University, Corvallis, OR 97331, USA; 2 Oregon Clinical and Translational Research Institute, Oregon Health and Sciences University, Portland, OR 97239, USA; 3 Dhaka Community Hospital Trust, Dhaka 1217, Bangladesh; 4 Harvard T.H. Chan School of Public Health, Department of Environmental Health, Harvard University, Boston, MA 02115, USA

**Keywords:** miscarriage, stillbirth, spontaneous abortion, fetal loss, neonatal mortality, groundwater, environmental policy, environmental epidemiology, drinking water, arsenic

## Abstract

(1) Background: Arsenic (As) is a common drinking water contaminant that is regulated as a carcinogen. Yet, As is a systemic toxicant and there is considerable epidemiological data showing As adversely impacts reproductive health. This study used data from a birth cohort in Bangladesh (2008–2011) to examine associations between drinking water As levels and reproductive outcomes. (2) Methods: Pregnant individuals (*n* = 1597) were enrolled at <16 weeks gestation and drinking water As was measured. Participants with live births (*n* = 1130) were propensity score matched to participants who experienced miscarriage (*n* = 132), stillbirth (*n* = 72), preterm birth (*n* = 243), and neonatal mortality (*n* = 20). Logistic regression was used to examine drinking water As recommendations of 50, 10, 5, 2.5, and 1 µg/L on the odds of adverse birth outcomes. (3) Results: The odds of miscarriage were higher for pregnant women exposed to drinking water ≥2.5 versus <2.5 µg As/L [adjusted odds ratio (OR) 1.90, 95% Confidence Interval (CI): 1.07–3.38)]. (4) Conclusions: These preliminary findings suggest a potential threshold where the odds of miscarriage increases when drinking water As is above 2.5 µg/L. This concentration is below the World Health Organizations and Bangladesh’s drinking water recommendations and supports the re-evaluation of drinking water regulations.

## 1. Introduction

Groundwater is an important source of potable water yet arsenic, a geogenic compound, contaminates groundwater in 107 countries [1]. Arsenic is a known human carcinogen and systemic toxicant. Currently, regulatory guidelines for arsenic in drinking water have been established using human health risk assessments that are specific for lung and bladder cancer. These regulatory guidelines have been adjusted over time as more epidemiological data was collected documenting the risk of cancer among people exposed to arsenic-contaminated drinking water. For example, the United States Environmental Protection Agency (US EPA) lowered the National Primary Drinking Water Regulation’s maximum contaminant level from 50 µg/L to 10 µg/L in October 2001 after analyses demonstrated the benefits of reducing bladder and lung cancer risks outweighed treatment costs based on epidemiological data collected in Taiwan [2,3,4,5]. The US enforcement of this new Arsenic Rule began in 2006 and surveillance studies show that it was effective at reducing arsenic exposure from public drinking water systems and that urinary arsenic levels declined among those people relied on public water systems [6,7]. Thus, environmental regulations that reduce maximum contaminant levels in regulated drinking water systems is effective at reducing arsenic exposure among whole populations in the US.

The World Health Organization (WHO) also based its provincial drinking water guideline at 10 µg/L based on arsenic’s carcinogenicity [8]. As additional health data documented the increased risk of skin cancer and other adverse effects associated with exposure to arsenic-contaminated drinking water, certain areas in the US and Denmark adopted even more protective drinking water regulatory recommendations. For example, New Jersey, US reduced their arsenic regulation in 2016 to 5 µg/L based on the National Research Council’s 1999 report on arsenic citing increased cancer risk and adverse health effects [9,10]. New Hampshire, US and Denmark also lowered their drinking water regulations to 5 µg/L citing skin cancer risks, cardiovascular disease risk, and risk of adverse birth and perinatal outcomes [11,12]. 

The human health effects of arsenic exposure have continued to be studied in populations around the world that rely on groundwater. Subsequently, there are considerable epidemiological data indicating that arsenic is a reproductive toxicant. However, it is unclear if drinking water regulatory standards established using cancer data are also protective against miscarriages, stillbirths, premature births, and neonatal mortality. These adverse reproductive outcomes are widespread and affect approximately 48 million pregnancies every year [13,14]. Bangladesh has made great strides in improving maternal and child health goals that stem from Bangladesh’s governing bodies signing the Millennium Development Goals (MDG) in the early 1990’s. Bangladesh made reductions in stillbirth and neonatal mortalities between 1990–2015, with one of the fastest reductions in stillbirth from 2000–2015 worldwide [15]. They were also able to reduce neonatal mortality by 40% from 2000–2015 [13,16]. Yet, even with these reductions in stillbirth and neonatal mortalities, Bangladesh ranks seventh in the world for stillbirths with a rate of 25.4 per 1000 live births [17]. 

The improvements Bangladesh made to these maternal child outcomes were largely based on efforts to address the major risks of adverse reproductive outcomes such as low nutritional status, limited birth spacing, and limited access to obstetric care [18,19]. However, there are multiple risk factors that can adversely impact reproductive success. For instance, neonatal mortality in Bangladesh typically arises from preterm birth, congenital abnormalities, asphyxia during birth, and infections due to pneumonia/sepsis [16]. Low birth weight (weight under 2500 g at birth) is also a risk factor for neonatal mortality as a product of preterm birth [20,21]. Yasmin et al. in Bangladesh, showed that low birth weight accompanied by preterm birth resulted in 75% of the neonatal mortality, of which 84% occurred 0 to 6 days post birth [20]. Studies that have examined the rate of miscarriage in Bangladesh have identified other risk factors including lower socioeconomic status, short birth spacing between pregnancies, and mothers aged over 35 years [19,22]. Furthermore, many epidemiological studies on arsenic-related health effects have been conducted in the past two decades. Several of these studies were conducted in Bangladesh because of the widespread arsenic exposure that resulted from switching the population’s source of drinking water from surface water to groundwater [23]. It was estimated that almost 57 million people were exposed to elevated arsenic levels in Bangladesh [24]. Recent remediation efforts have made great strides in reducing the country’s exposure to arsenic by mitigating the source including labelling and stop usage of contaminated wells, community or single occupancy filtration systems, and installing entirely new wells [25]. Even with relief efforts, about 13% of drinking water samples tested had elevated levels of arsenic above 50 µg/L, which is the current drinking water recommendation in Bangladesh [25]. Thus, re-examining the regulatory level for arsenic in drinking water could yield further improvements in maternal child health.

Using an established prospectively enrolled cohort, we examined the associations between arsenic exposure at different regulatory cutoff levels and adverse pregnancy outcomes in Bangladesh. The regulatory level cut offs were used to compare risks of neonatal mortality, stillbirth, miscarriage, and preterm birth between different arsenic exposure groups. Propensity score methodology was used to control for confounding by matching pregnant women on predictors of arsenic exposure status. We hypothesized that the odds of neonatal mortality, stillbirth, miscarriage, and preterm birth would be higher for pregnant women who were exposed to arsenic above 10 µg/L compared to those below this WHO and US EPA regulatory cut off level. Thresholds below these guidelines were also examined to understand if there was a potential threshold below which the odds of adverse pregnancy outcomes remain stable.

## 2. Materials and Methods

### 2.1. Study Population

From 2008–2011, mothers were recruited in two study areas (Sirajdikhan and Pabna Sadar Upazilas) of Bangladesh in partnership with Dhaka Community Hospital Trust (DCHT) (Figure S1). The study was designed to examine the exposure-response relationship between arsenic and birthweight and the design has been described elsewhere [26,27]. Briefly, these regions were chosen because groundwater surveys indicated that arsenic was present in the groundwater across a wide range of concentrations which would facilitate examining an exposure-response relationship. Additionally, DCHT operated a rural health care clinic in these areas that included a network of trained midwives. Clinic staff and midwives told pregnant women in the clinic catchment areas about the study. Recruitment occurred at the clinics and represented a convenience sample. Participants were eligible if they were ≥18 years of age, enrolled with a singleton ultrasound at pregnancy ≤16 weeks gestation, primary source of water was a ground water tube well, stayed in the same location for the length of pregnancy, use of the prenatal care from DCHT, and agreed to birth child at DCHT or by a DCHT-trained midwife [26]. Clinical visits and medical care were provided by DCHT trained medical staff and field technicians. Mothers provided data and socio-demographic information using structured questionnaires at <16 weeks gestation, during monthly follow-up home visits, at 28 weeks gestational age, at birth, and one-month post-partum (if applicable). This study was approved by the Human Research Committees/Institutional Review Boards at Oregon State University (OSU), Harvard TH Chan School of Public Health, and DCHT. Informed consent was given by all participants before the collection of data for the study.

Participants were included in this analysis if they had an ultrasound at the time of enrollment (<16 weeks gestation), had a singleton pregnancy, and provided a drinking water sample that was measured for arsenic. Participants with missing data at the time of enrollment were excluded because propensity score matching requires complete data. Of the 1613 pregnant women enrolled in the original cohort, 4 women were excluded for not having an ultrasound at enrollment, 2 women were excluded for lacking exposure data, 8 women were excluded for having twins, and 2 women were excluded for missing enrollment data. 

### 2.2. Fetal Loss, Preterm Birth, and Neonatal Mortality

Fetal loss was defined as a fetus that was not alive outside of the womb. Miscarriage was the loss of a fetus before a viable birth (*n* = 132) and stillbirth was the loss of a fetus during a viable birth period (including preterm, *n* = 72). Neonatal mortality was determined by mortality of newborn within the first 30 days after live birth from womb (*n* = 20). Preterm birth was defined as a viable birth < 37 weeks gestation and lived past one-month post-partum (*n* = 243). Gestational age was determined at enrollment via ultrasonography at the first visit and calculated by mean gestational sac diameter at 4-6 weeks or by crown-rump length at 7–16 weeks. Ultrasounds were conducted with two technicians for each participant and verified by an obstetrician at DCH. Trained field staff confirmed all neonatal mortalities with the parents within the first 30 days of life for *n* = 1150 live births. 

### 2.3. Exposure Assessment: Drinking Water Arsenic

Drinking water samples were collected from the participants primary drinking water source which was a groundwater tube well and arsenic concentrations were measured using inductively coupled plasma mass spectrometry. Sample collection methods are described elsewhere [28,29]. Briefly, water was collected at the time of enrollment for all women. Samples were preserved to pH < 2 using ultrapure nitric acid, kept at room temperature, and measured for arsenic following EPA methods 200.8. The limit of detection (LOD) for arsenic was 0.5 μg/L and there were 304 samples below the LOD. The samples below the LOD were given a value of 0.5 μg/L divided by the square root of 2.

### 2.4. Covariates

Covariates were chosen *a priori* based on their associations with arsenic and fetal loss [26,30,31]. These included maternal age (continuous), self-reported highest level of maternal education (illiterate/able to write name; primary education; secondary education and higher education), cook fuel type within home (wood or natural gas versus dung or cross-residue), body mass index (BMI) derived from the weight at time of enrollment (kg) divided by height^2^ (m^2^) (continuous), monthly family income reported by the financial provider of the mother (Taka: >3000, 3001–4000, 4001–5000, 5000+), clinic location (Pabna or Sirajdikhan), previous miscarriage or stillbirth if not first child (yes/no), and parity (1st child or 2nd and greater). Models for neonatal mortality and preterm birth were controlled for the above covariates as well as: birth gestational age (only controlled in neonatal mortality, in weeks), birth type (vaginal or cesarean), sex of child (male or female), and birthweight of child (kg).

### 2.5. Statistical Approaches

Descriptive statistics (means, medians, and frequencies) were calculated for continuous variables. Study participants were matched using propensity scores. Rosenbaum and Rubin [32] developed propensity score matching to create comparative groups with respect to measured explanatory variables for observational studies. We estimated propensity scores with optimal full matching using logistic regression via the R package MatchIt [33], where the binary dependent variable was whether or not drinking water arsenic concentration was about a threshold (e.g., 10 μg/L) and the independent variables were the covariates mentioned above based on outcome. Through numeric review of standardized mean differences and graphical inspections of empirical quantile-quantile plots, the matching yielded adequate balance. Nearest neighbor and optimal pair matching also were considered, but optimal full matching effectively produced balanced matches (Figure S2 and Supplemental Table S3). Matched samples were analyzed using conditional logistic regression to estimate the association between level of arsenic exposure and adverse birth outcomes. Matching produced dependent data and therefore, the logistic regression incorporated weights and a cluster-robust variance of estimated regression coefficients was used to calculate confidence intervals. Multicollinearity was assessed using variance inflation factors, which were all below 5. Statistical significance was set at α = 0.05, interval estimates were computed using a 95% confidence level, and all analyses were performed in R version 4.0.3.

Sensitivity analyses evaluated the robustness of our findings for the adverse outcome of miscarriage. Specifically, results from unadjusted and adjusted logistic regression analyses of the unmatched data were compared to results from full and nearest-neighbor propensity score matched analyses. 

## 3. Results

**Figure 1:**
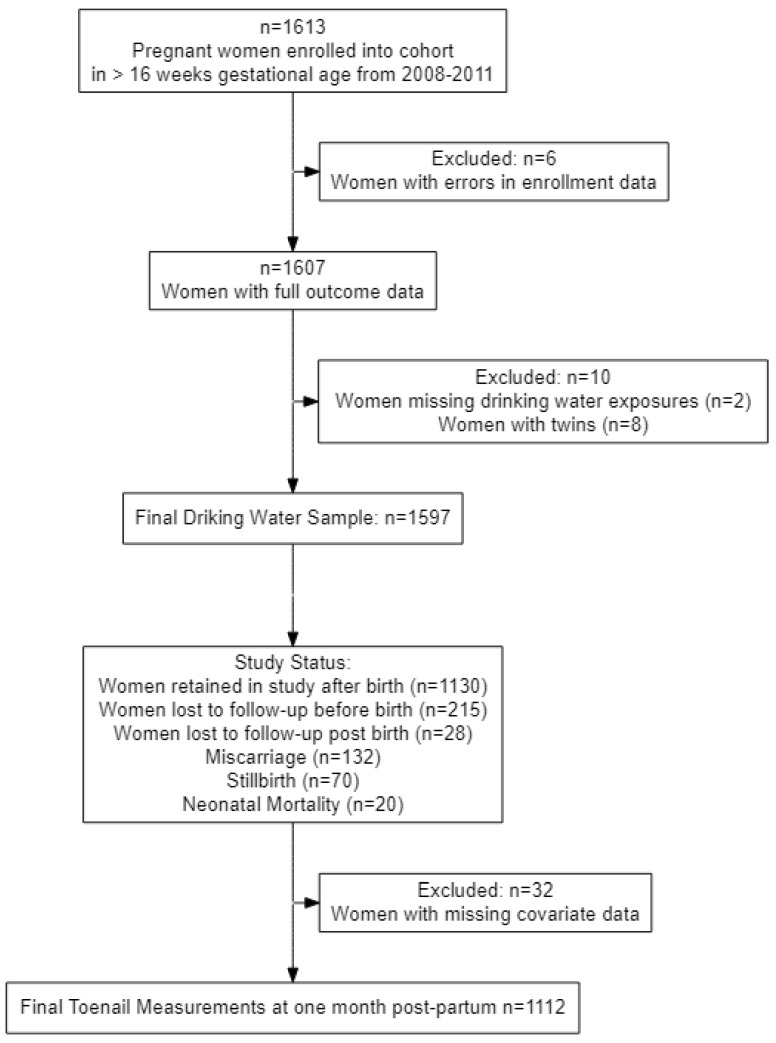
Study population for analysis.

Of the 1597 women who met the inclusion criteria of this analysis; 1130 women had live births, 132 women experienced a miscarriage, 72 women experienced a stillbirth, and 20 infants were lost due to neonatal mortalities one month after birth. Within the live births and neonatal mortalities there were 243 preterm births. Additionally, 215 women withdrew or were lost to follow up prior to birth, and 28 women withdrew from the study after birth. Figure 1 describes the final study sample (*n* = 1354). The characteristics of mothers with and without adverse birth outcomes are presented in Table 1. Women who experienced miscarriage tended to be in the higher income taka brackets (4000–5000 and 5000+) and were in Sirajdikhan research area. Participants who experienced stillbirth fetal loss tended to have the lower income taka brackets (>3000 and 3000–4000) and located in Sirajdikhan research area. In cases of neonatal mortality, mothers tended to have preterm birth of a viable birth before 37 weeks gestation and lower birth weight for the child. Mean drinking water manganese were similar for women with live births and adverse birth outcomes, while the median levels for those who experienced stillbirth tended to be lower. About 22% of the participant’s used water wells that contained arsenic above 50 µg/L (range: 0.5–1400 µg/L). Approximately one third (38%) of drinking water arsenic levels were above 10 µg/L, 45% were above 5 µg/L, 49% were above 2.5 µg/L, and 72% were above 1 µg/L. 

### 3.1. Miscarriage and Drinking Water Arsenic 

We used optimal full propensity score matched samples to compare different drinking water arsenic levels against adverse pregnancy outcomes (Table 2). These analyses showed that there was a potential threshold at 2.5 µg/L. The adjusted odds of miscarriage were 90% (7–238%) higher among pregnant women exposed to ≥2.5 µg/L arsenic in drinking water compared to those exposed to <2.5 µg/L. Pregnant women exposed to ≥5 µg/L of arsenic in drinking water had 2.73 (1.49–5.02) times the adjusted odds of miscarriage relative to those exposed to <5 µg/L. The odds of miscarriage were not significantly different at the higher cutoff levels of 10 µg/L (adjusted odds ratio: 1.18, 0.60–2.30) and 50 µg/L (adjusted odds ratio: 0.66, 0.33–1.33). Our analysis did not find a specific threshold of drinking water arsenic levels which the odds of neonatal mortality, stillbirth, or preterm birth were decreased or increased. 

### 3.2. Miscarriage Sensitivity Analysis

Sensitivity analyses were conducted to determine the robustness of the drinking water arsenic threshold on miscarriage between different modeling parameters (unmatched, full matching, and nearest neighbor matching). Comparisons between model types, matching diagnostics, and descriptive statistics between matched and unmatched cohorts is tabled in the Supplemental Material. The unmatched sample was larger because matching resulted in reducing the control group by 10 participants and the cases by 15 participants because of missing covariate data for household income and cook fuel type. Besides the reduction in sample size, there were little differences in the distribution of covariates between the unmatched and matched samples (Table S1). Analyses for drinking water arsenic threshold comparisons for those participants who experienced miscarriage were broken down and compared by unmatched crude logistic regression, unmatched adjusted logistic regression, nearest neighbor, and full propensity score matching methods in Supplementary Table S2. Generally, we found that the unmatched unadjusted logistic regression models demonstrated different effect sizes than the unmatched adjusted logistic regression. Our analyses used different methods of propensity score matching (e.g., nearest neighbor and optimal full matching). Standard mean differences reported in Supplementary Table S3 showed that balance was achieved by optimal full matching rather than nearest neighbor matching. 

## 4. Discussion

This preliminary analysis of the effect of arsenic exposure in drinking water and adverse birth outcomes observed an increased risk of miscarriage in this Bangladesh cohort when drinking water arsenic concentrations were above 2.5 µg/L. This risk dissipated at higher concentrations which could indicate a non-linear or perhaps U-shaped exposure-response relationship between drinking water arsenic concentrations and miscarriage. Alternatively, the attenuation of the risk of miscarriage at higher concentrations could also be a function of the underlying sample distribution. However, this finding is important because it indicates that the Bangladesh drinking water regulatory levels of 50 µg/L (and the WHO 10 µg/L guideline) was not protective against an elevated risk of miscarriage. Our analysis did not find a protective drinking water level for neonatal mortality, stillbirth, or preterm birth. These adverse outcomes were rare in this population-based prospective cohort of healthy women which resulted in a relatively limited sample size. Thus, our findings should be considered preliminary with larger studies needed to confirm our findings.

Our analysis focused on reproductive health outcomes which have been shown to be influenced by arsenic and suggests that pregnant women are a particularly vulnerable sub-group of the population that may not be protected by current drinking water arsenic regulations. Currently, the human health risk assessment process that yields regulatory drinking water recommendations in Bangladesh is based on the older US EPA cost–benefit analysis that did not include epidemiological data from Taiwan. Additionally, the WHO and US drinking water regulations are based on a cost–benefit that only included the risk for lung and bladder cancer [9]. While the balance between health benefit and economic reduction is delicate as the investment in filtration and water distribution can be burdensome on communities [34] there is ample epidemiological data documenting that arsenic exposure increases the risk of non-cancer health outcomes. Arsenic is known to cross the placenta from mother to fetus [35], although it is not transferred from mother to child via breast feeding [36]. Numerous epidemiological studies in multiple populations have shown that prenatal arsenic is associated with a greater risk of preterm birth, low birth weight, spontaneous abortion, and fetal loss [26,37,38,39,40]. In a systematic review and meta-analysis by Quansah et al., elevated levels of drinking water arsenic exposure (≥50 µg/L) was associated with increased risks of adverse pregnancy outcomes such as spontaneous abortion (OR: 1.98, 95% CI: 1.27–3.10), reduction in birth weight (β: −53.2 g, 95% CI: −94.9, −11.4), and infant mortality (OR: 1.35, 95% CI: 1.12–1.62) [41], and neonatal mortality (OR: 1.51, 95% CI: 1.28–1.78) [38,39,41,42,43,44]. Of the five studies from 2000-2015 of neonatal mortality, three of the studies were based in Bangladesh or West Bengal, India, while the other two were in Mongolia and Chile. There is growing evidence for arsenic’s toxicity at lower levels of drinking water exposure (<10 µg/L). Within our research team, Ahmed et al. showed for low-level drinking water arsenic exposure, a per unit increase in natural log arsenic resulted in a hazard ratio of fetal loss/neonatal mortality of 1.35 (95% CI: 1.08–1.69) between weeks 25–28 of gestation [40]. As a potential mechanism in adverse pregnancy outcomes with altered immune systems, this cohort also saw a reduction in immune response to vaccinations of diphtheria and tetanus with low-level drinking water arsenic exposure [28]. There is a gap in knowledge of low-level arsenic exposure during pregnancy and risk of fetal loss, preterm birth, and neonatal mortality within Bangladesh.

Our findings are consistent with other research groups in Bangladesh who have shown that drinking water arsenic exposures are associated with adverse birth outcomes [41,45]. Most of the studies which have reviewed adverse birth outcomes focused on exposures at or greater than 50 µg/L, leaving few studies comparing lower exposures with adverse birth outcomes. Research studies tended to use values of >10 µg/L as referent groups comparing to high exposures of drinking water arsenic [41,45]. A study of low to moderate drinking water arsenic exposures in Romania did not show any association with miscarriage (OR = 0.98, 95% CI: 0.96–1.01, *n* = 150), a sample size of miscarriages similar to our study [46]. Within this maternal-child cohort in Bangladesh, a common exposure pathway for arsenic is from both contaminated drinking water and diet [47,48,49], which could increase a mother’s internal arsenic exposure with higher toxicity. Previous work within this cohort has demonstrated the moderate correlation between drinking water arsenic and internal toenail measurements alluding to drinking water arsenic being one of the primary sources for the mother’s exposure [26]. Outside of pregnancy outcomes, neurological studies in the US have shown low arsenic exposure between 5–10 ug/L was associated with 6.09 points lost on Full Scale IQ points (95% CI: −9.99–−2.19) and other neurological parameters [50] which points to long term consequences from prenatal arsenic exposure. Experimental studies also show that arsenic is toxic to fetal development and survival of newborn pups [51,52]. Specifically, arsenic influences fetal programming and development via increased telomerase activity, diminishing immune development in mother, increasing oxidative stress, reducing hormone activities such as estrogen and glucocorticoids, modulating ovarian development, and changes in DNA methylation [53,54,55,56,57,58,59]. 

Propensity score matching was used within this cohort to address residual confounding. As mentioned previously, our non-matched logistic regression models did not show significant odds of miscarriage compared to those regressions using propensity score matching. The one significant predictor in our models with arsenic exposure was location of clinic for either Pabna or Sirajdikhan. While Bangladesh tends to have a fairly homogenous culture and practices, especially among these more rural areas, they do differ. Pabna is a more rural community with more farmers while Sirajdikhan is more peri-urban and closer to the capital city of Dhaka. Exposure profiles also differ between these two locations beyond arsenic. The use of propensity score matching by full matching allowed for more specific comparisons of location and exposure to measure odds of miscarriage in our models. Additionally, full matching allowed for more flexibility of matching where all data was used and matching at least one control to each case. Propensity score matching is not without its downsides. Researchers pointed out that “greedy” propensity score matching such as nearest-neighbor can exclude many data points but also reduce the bias of having lessor matches of controls in cohort studies [60]. Our approach of using full matching used most of the data and allowed for more flexibility between matching of cases to controls, where we saw the best balance between the exposed and non-exposed groups [61,62]. Our use of full matching allowed for lowered standard mean differences and better graphical observations while reducing the variances (while using robust variance estimators) and increasing effect sizes [63]. The exposure of arsenic was common within our cohort, as we did not specify a “treated” and “untreated” grouping, rather based on a cut-off value of exposure [64]. 

It is also worth noting that our population-based sample included a largely healthy birth cohort of pregnant women. There was no tobacco use and very little preexisting conditions such as underweight or overweight, diabetes, or hypertension. Very few women were hypertensive during pregnancy, developed gestational diabetes, or developed pre-eclampsia. Our exposure assessment utilized individual level drinking water arsenic measurements. This does introduce some exposure misclassification because it does not include dietary intake. However, previous studies in the cohort demonstrated the high correlation between drinking water and internal toenail arsenic exposures, identifying drinking water as one of the main sources of exposure [65]. There is potential for alternative sources of arsenic from diet or lifestyle factors to influence the body burden of arsenic which was not explored in this study.

There are several limitations in this study. Due to low number of neonatal mortality cases (*n* = 20), we were only able to compare proportions of matched cases and controls in association models. Future studies with larger sample sizes of adverse birth outcomes such as neonatal mortality and stillbirth could possibly see a threshold value in the examples we used. We were unable to use toenail arsenic exposures in our study as individuals that experienced miscarriage or stillbirth did not have measurements taken after birth. The use of propensity scores allowed our results to be significant with an alpha of 0.05, where traditional adjusted logistic regression models were null. The direction of the effect size was similar between the two methods, the use of propensity scores creates dependent study samples which are not comparable to the entire population. There is a chance that k:1 propensity score matching could increase bias of the estimation with additional matches beyond 1:1, yet full matching relaxes that assumption [60]. Additionally, with the use of propensity scores, we were not able to review a continuous exposure of arsenic, only between a cut off value which lowers the ability to see specific changes in odds of an outcome. Our sample was comprised almost entirely of Muslim women who do not partake in smoking or alcohol consumption. On the advice of our community advisory boards, questions regarding these questions were not included in our surveys because they would be unnecessary and offensive to the participants. However, our analyses did examine exposures from burning biomass with cookstove type and included as a covariate in our models.

## 5. Conclusions

Women during pregnancy are vulnerable to environmental contamination through ingestion of drinking water and this vulnerability is not currently taken into consideration when establishing drinking water guidelines. We observed that the odds of miscarriage increased when drinking water arsenic was above 2.5 µg/L, a concentration that is well below the current WHO’s provisional drinking water guideline and Bangladesh’s drinking water recommendations. Our findings are preliminary because of the relatively small number of adverse birth outcomes observed in this cohort but it helps support the understanding of low-level arsenic exposures and associations with adverse pregnancy outcomes, not just at higher levels of over 50 µg/L. While pregnant women are still exposed to unsafe levels of arsenic around the world, clinicians and providers need to be aware of all levels of arsenic exposure to reduce adverse effects and support healthy pregnancies. 

## Figures and Tables

**Table 1:** Characteristics of Bangladesh Cohort by Adverse Pregnancy Outcome (*n* = 1597).

	Live Births (*n* = 1130)	Miscarriage (*n* = 132)	Stillbirth (*n* = 72)	Neonatal Mortality (*n* = 20)	Overall (*n* = 1597)	*p*-Value **
**Age (years)**						0.6
Mean (SD)	23.0 (4.22)	23.1 (4.45)	22.2 (3.76)	23.2 (4.02)	22.9 (4.19)	
Median [Min, Max]	22.0 [18.0, 41.0]	22.0 [18.0, 35.0]	21.0 [18.0, 35.0]	23.0 [18.0, 33.0]	22.0 [18.0, 41.0]	
**BMI (Kg/m^2^)**						0.4
Mean (SD)	20.5 (3.23)	20.8 (3.18)	20.2 (3.04)	19.8 (2.69)	20.5 (3.20)	
Median [Min, Max]	20.0 [13.4, 36.0]	20.4 [12.6, 29.0]	20.1 [15.3, 30.2]	19.3 [14.3, 25.4]	20.1 [12.3, 36.0]	
**Education**						0.1
No school attendance	159 (14.1%)	24 (18.2%)	13 (18.1%)	6 (30.0%)	236 (14.8%)	
Primary School	365 (32.3%)	45 (34.1%)	29 (40.3%)	7 (35.0%)	540 (33.8%)	
Secondary School and Higher Education	606 (53.6%)	63 (47.7%)	30 (41.7%)	7 (35.0%)	821 (51.4%)	
**Monthly Household Income (Taka)**						0.04
<3000	188 (16.6%)	11 (8.3%)	19 (26.4%)	5 (25.0%)	253 (15.8%)	
3001-4000	298 (26.4%)	25 (18.9%)	16 (22.2%)	7 (35.0%)	394 (24.7%)	
4001-5000	336 (29.7%)	43 (32.6%)	17 (23.6%)	4 (20.0%)	493 (30.9%)	
5001+	301 (26.6%)	38 (28.8%)	14 (19.4%)	4 (20.0%)	425 (26.6%)	
Missing	7 (0.6%)	15 (11.4%)	6 (8.3%)	0 (0%)	32 (2.0%)	
**Cook Fuel Type**						0.1
Clean Fuel	778 (68.8%)	86 (65.2%)	38 (52.8%)	15 (75.0%)	1090 (68.3%)	
Less Clean Fuel	346 (30.6%)	46 (34.8%)	34 (47.2%)	5 (25.0%)	499 (31.2%)	
Refused	3 (0.3%)	0 (0%)	0 (0%)	0 (0%)	5 (0.3%)	
Missing	3 (0.3%)	0 (0%)	0 (0%)	0 (0%)	3 (0.2%)	
**Risk of subsequent miscarriage/stillbirth**						0.8
First Born or None Previous	922 (81.6%)	105 (79.5%)	59 (81.9%)	15 (75.0%)	1312 (82.2%)	
Previous Miscarriage/Stillbirth	208 (18.4%)	27 (20.5%)	13 (18.1%)	5 (25.0%)	285 (17.8%)	
**Number of previous births**						0.8
First Born	536 (47.4%)	68 (51.5%)	37 (51.4%)	10 (50.0%)	768 (48.1%)	
2nd or Greater Born	594 (52.6%)	64 (48.5%)	35 (48.6%)	10 (50.0%)	829 (51.9%)	
**Clinic Location**						0.02
Pabna	555 (49.1%)	48 (36.4%)	31 (43.1%)	12 (60.0%)	726 (45.5%)	
Sirajdikhan	575 (50.9%)	84 (63.6%)	41 (56.9%)	8 (40.0%)	871 (54.5%)	
**Preterm Birth (> 37 Weeks Gestation)**						<0.001
No	887 (78.5%)	--	--	9 (45.0%)	918 (57.5%)	
Yes	243 (21.5%)	--	--	11 (55.0%)	260 (16.3%)	
Missing	--	132 (100%)	72 (100%)	--	419 (26.2%)	
**Gestational Age (Weeks)**						0.009
Mean (SD)	38.0 (1.85)	--	--	34.8 (5.08)	37.9 (2.00)	
Median [Min, Max]	38.0 [29.0, 42.0]	--	--	35.5 [22.0, 40.0]	38.0 [22.0, 42.0]	
Missing	--	132 (100%)	72 (100%)	--	419 (26.2%)	
**Child’s Weight at Birth (kg)**						0.01
Mean (SD)	2.84 (0.402)	--	--	2.52 (0.760)	2.84 (0.409)	
Median [Min, Max]	2.86 [0.800, 4.80]	--	--	2.50 [1.40, 4.60]	2.86 [0.800, 4.80]	
Missing	--	132 (100%)	72 (100%)	--	419 (26.2%)	
**Child’s Sex at Birth**						0.5
Male	574 (50.8%)	--	--	12 (60.0%)	598 (37.4%)	
Female	556 (49.2%)	--	--	8 (40.0%)	580 (36.3%)	
Missing	--	132 (100%)	72 (100%)	--	419 (26.2%)	
**Type of Birth**						0.06
Cesarean	404 (35.8%)	--	--	3 (15.0%)	413 (25.9%)	
Vaginal	726 (64.2%)	--	--	17 (85.0%)	765 (47.9%)	
Missing	--	132 (100%)	72 (100%)	--	419 (26.2%)	
**Drinking Water Manganese (µg/L)**						0.5
Mean (SD)	722 (704)	708 (715)	643 (671)	734 (630)	728 (729)	
Median [Min, Max]	590 [0.500, 4720]	620 [1.00, 3500]	490 [1.00, 2600]	670 [1.50, 2700]	590 [0.500, 5300]	
**Drinking Water Arsenic cut off 1 µg/L**						0.4
<1 µg/L	312 (27.6%)	36 (27.3%)	20 (27.8%)	2 (10.0%)	442 (27.7%)	
≥1 µg/L	818 (72.4%)	96 (72.7%)	52 (72.2%)	18 (90.0%)	1155 (72.3%)	
**Drinking Water Arsenic cut off 2.5 µg/L**						0.2
<2.5 µg/L	576 (51.0%)	73 (55.3%)	43 (59.7%)	7 (35.0%)	845 (52.9%)	
≥2.5 µg/L	554 (49.0%)	59 (44.7%)	29 (40.3%)	13 (65.0%)	752 (47.1%)	
**Drinking Water Arsenic cut off 5 µg/L**						0.3
<5 µg/L	624 (55.2%)	78 (59.1%)	46 (63.9%)	9 (45.0%)	915 (57.3%)	
≥5 µg/L	506 (44.8%)	54 (40.9%)	26 (36.1%)	11 (55.0%)	682 (42.7%)	
**Drinking Water Arsenic cut off 10 µg/L**						0.3
<10 µg/L	699 (61.9%)	88 (66.7%)	50 (69.4%)	10 (50.0%)	1014 (63.5%)	
≥10 µg/L	431 (38.1%)	44 (33.3%)	22 (30.6%)	10 (50.0%)	583 (36.5%)	
**Drinking Water Arsenic cut off 50 µg/L**						0.4
<50 µg/L	886 (78.4%)	108 (81.8%)	61 (84.7%)	14 (70.0%)	1271 (79.6%)	
≥50 µg/L	244 (21.6%)	24 (18.2%)	11 (15.3%)	6 (30.0%)	326 (20.4%)	

** *p*-values from chi-squared or Kruskal–Wallis tests were calculated between levels each covariate or exposure and adverse birth outcome.

**Table 2:** Odds of adverse pregnancy risk based on cut-off regulatory/recommendation values for drinking water arsenic for women in Bangladesh.

		Goal or Regulatory Cut-off of Arsenic (µg/L)									
		1 µg/L		2.5 µg/L		5 µg/L		10 µg/L		50 µg/L	
		*Below*	*Above*	*Below*	*Above*	*Below*	*Above*	*Below*	*Above*	*Below*	*Above*
Miscarriage ^†^ (*n* = 132)	Yes	6	111	70	47	74	43	83	34	99	18
	No	69	1051	569	551	617	503	691	429	878	242
	Odds Ratio	1.09 (0.69–1.73)		**1.90 (1.07–3.38)**		**2.73 (1.49–5.02)**		1.18 (0.60–2.30)		0.66 (0.33–1.33)	
Stillbirth ^†^ (*n* = 72)	Yes	20	46	41	25	42	24	46	20	56	10
	No	309	811	569	551	617	503	691	429	878	242
	Odds Ratio	0.72 (0.36–1.43)		1.21 (0.60–2.43)		0.60 (0.28–1.30)		0.86 (0.44–1.71)		0.70 (0.31–1.59)	
Preterm Birth * (*n* = 260)	Yes	44	211	71	184	81	174	112	143	165	90
	No	258	614	494	378	533	339	577	295	714	158
	Odds Ratio	1.06 (0.57–1.99)		1.06 (0.56–1.97)		1.44 (0.91–2.31)		1.04 (0.67–1.61)		1.43 (0.93–2.20)	
Neonatal Mortality (*n* = 20) *	Yes	2	18	7	13	9	11	10	10	14	6
	No	300	803	558	545	605	498	679	424	864	239
	Odds Ratio	3.36 (0.78–14.58)		1.90 (0.75–4.8)		1.48 (0.61–3.61)		1.60 (0.66–3.88)		1.55 (0.59–4.0706)	

^†^ Miscarriage and stillbirth propensity score matching and model covariates: age, BMI, income, education, household cook fuel type, location of clinic, risk of previous miscarriage or stillbirth if not first child, parity, and drinking water manganese. * Neonatal mortality and preterm birth propensity score matching and model covariates: age, BMI, income, education, household cook fuel type, location of clinic, birth gestational age (neonatal mortality only), birth either vaginal or cesarean, parity, sex of child, birthweight of child (kg), risk of previous miscarriage or stillbirth if not first child, drinking water manganese, and blood lead. Sample sizes for each adverse birth outcome: Miscarriage *n* = 1237, Stillbirth *n* = 1186, Preterm Birth *n* = 1127, Neonatal Mortality *n* = 1123.

## Supplementary Materials

The following supporting information can be downloaded at: https://www.mdpi.com/article/10.3390/toxics10100600/s1.

## References

[1] Shaji E., Santosh M., Sarath K.V., Prakash P., Deepchand V., Divya B.V. (2021). Arsenic contamination of groundwater: A global synopsis with focus on the Indian Peninsula. Geosci. Front..

[2] US EPA (2000). Arsenic in Drinking Water Rule Economic Analysis.

[3] Chen C.J., Chuang Y.C., Lin T.M., Wu H.Y. (1985). Malignant neoplasms among residents of a blackfoot disease-endemic area in Taiwan: High-arsenic artesian well water and cancers. Cancer Res..

[4] Chen C.J., Wu M.M., Lee S.S., Wang J.D., Cheng S.H., Wu H.Y. (1988). Atherogenicity and carcinogenicity of high-arsenic artesian well water. Multiple risk factors and related malignant neoplasms of blackfoot disease. Arteriosclerosis.

[5] Chen C.J., Chen C.W., Wu M.M., Kuo T.L. (1992). Cancer potential in liver, lung, bladder and kidney due to ingested inorganic arsenic in drinking water. Br. J. Cancer.

[6] Welch B., Smit E., Cardenas A., Hystad P., Kile M.L. (2018). Trends in urinary arsenic among the U.S. population by drinking water source: Results from the National Health and Nutritional Examinations Survey 2003–2014. Environ. Res..

[7] Foster S.A., Pennino M.J., Compton J.E., Leibowitz S.G., Kile M.L. (2019). Arsenic Drinking Water Violations Decreased across the United States Following Revision of the Maximum Contaminant Level. Environ. Sci. Technol..

[8] World Health Organization (2011). Arsenic in Drinking Water: Background Document for Development of WHO Guidelines for Drinking-water Quality. WHO/SDE/WSH/03.04/75/Rev/1. https://www.who.int/water_sanitation_health/dwq/chemicals/arsenic.pdf?ua=1.

[9] (2001). Report NRC (US) S to U the 1999 A in DW. Introduction Arsenic in Drinking Water: 2001 Update.

[10] NJDEP New Jersey Department of Environmental Protection. https://www.state.nj.us/dep/dsr/arsenic/guide.htm.

[11] NH DES (2018). Review of the Drinking Water Maximum Contaminant Level (MCL) and Ambient Groundwater Quality Standard (AGQS) for Arsenic. New Hamps. Dep. Environ. Serv..

[12] Ministry of Environment and Food of Denmark (2019). Bekendtgørelse om Vandkvalitet og Tilsyn med Vandforsyningsanlæg. BEK no. 802. https://www.vandcenterdjurs.dk/media/1133/gaeldende-drikkevandsbekendtgoerelse-28-10-2019.pdf.

[13] Neonatal Mortality UNICEF DATA. https://data.unicef.org/topic/child-survival/neonatal-mortality/.

[14] USAID (2014). USAID Maternal Health Strategy, 2014–2020. Toward Ending Preventable Maternal Mortality. https://www.mchip.net/sites/default/files/mchipfiles/USAID%20MH%20Strategy%20Apr.22.14.pdf.

[15] Lawn J.E., Blencowe H., Waiswa P., Amouzou A., Mathers C., Hogan D., Draper E.S. (2016). Stillbirths: Rates, risk factors, and acceleration towards 2030. Lancet.

[16] Lawn J.E., Cousens S., Zupan J. (2005). Lancet Neonatal Survival Steering Team. 4 million neonatal deaths: When? Where? Why?. Lancet.

[17] Blencowe H., Cousens S., Jassir F.B., Say L., Chou D., Mathers C., Hogan D., Shiekh S., Qureshi Z., You D. (2016). National, regional, and worldwide estimates of stillbirth rates in 2015, with trends from 2000: A systematic analysis. Lancet Global Health.

[18] Lawn J.E., Yakoob M.Y., Haws R.A., Soomro T., Darmstadt G.L., Bhutta Z.A. (2009). 3.2 million stillbirths: Epidemiology and overview of the evidence review. BMC Pregnancy Childbirth.

[19] Nonyane B.A.S., Norton M., Begum N., Shah R.M., Mitra D.K., Darmstadt G.L., Baqui A.H., Projahnmo Study Group in Bangladesh, for the Projahnmo Study Group in Bangladesh (2019). Pregnancy intervals after stillbirth, neonatal death and spontaneous abortion and the risk of an adverse outcome in the next pregnancy in rural Bangladesh. BMC Pregnancy Childbirth.

[20] Yasmin S., Osrin D., Paul E., Costello A. (2001). Neonatal mortality of low-birth-weight infants in Bangladesh. Bull World Health Organ..

[21] McCormick M.C. (1985). The contribution of low birth weight to infant mortality and childhood morbidity. N. Engl. J. Med..

[22] Rashid H., Ma E., Ferdous F., Ekström E.C., Wagatsuma Y. (2017). First-trimester fetal growth restriction and the occurrence of miscarriage in rural Bangladesh: A prospective cohort study. PLoS ONE.

[23] Caldwell B.K., Caldwell J.C., Mitra S.N., Smith W. (2003). Searching for an optimum solution to the Bangladesh arsenic crisis. Soc. Sci. Med..

[24] Arsenic Contamination of Groundwater in Bangladesh|British Geological Survey (BGS). http://www.bgs.ac.uk/arsenic/bangladesh/.

[25] Johnston R. (2011). Bangladesh National Drinking Water Quality Survey of 2009.

[26] Kile M.L., Rodrigues E.G., Mazumdar M., Dobson C.B., Diao N., Golam M., Quamruzzaman Q., Rahman M., Christiani D.C. (2014). A prospective cohort study of the association between drinking water arsenic exposure and self-reported maternal health symptoms during pregnancy in Bangladesh. Environ. Health.

[27] Rodrigues E.G., Kile M., Dobson C., Amarasiriwardena C., Quamruzzaman Q., Rahman M., Golam M., Christiani D.C. (2015). Maternal–infant biomarkers of prenatal exposure to arsenic and manganese. J. Expo. Sci. Environ. Epidemiol..

[28] Welch B.M., Branscum A., Ahmed S.M., Hystad P., Smit E., Afroz S., Megowan M., Golam M., Hasan O.S.I., Rahman M.L. (2019). Arsenic exposure and serum antibody concentrations to diphtheria and tetanus toxoid in children at age 5: A prospective birth cohort in Bangladesh. Environ. Int..

[29] Welch B.M., Branscum A., Geldhof G.J., Ahmed S.M., Hystad P., Smit E., Afroz S., Megowan M., Golam M., Sharif O. (2020). Evaluating the effects between metal mixtures and serum vaccine antibody concentrations in children: A prospective birth cohort study. Environ. Health.

[30] Rahman A., Vahter M., Smith A.H., Nermell B., Yunus M., El Arifeen S., Persson L., Ekström E.-C. (2009). Arsenic Exposure during Pregnancy and Size at Birth: A Prospective Cohort Study in Bangladesh. Am. J. Epidemiol..

[31] Steinmaus C., Yuan Y., Bates M.N., Smith A.H. (2003). Case-Control Study of Bladder Cancer and Drinking Water Arsenic in the Western United States. Am. J. Epidemiol..

[32] Rosenbaum P.R., Rubin D.B. (1985). Constructing a Control Group Using Multivariate Matched Sampling Methods That Incorporate the Propensity Score. Am. Stat..

[33] Ho D.E., Imai K., King G., Stuart E.A. (2011). MatchIt: Nonparametric Preprocessing for Parametric Causal Inference. J. Stat. Softw..

[34] Cho Y., Easter K.W., Konishi Y. (2010). Economic Evaluation of the New U.S. Arsenic Standard for Drinking Water: A Disaggregate Approach. Water Resources Research. https://agupubs.onlinelibrary.wiley.com/doi/abs/10.1029/2009WR008269.

[35] Eastman N.J. (1931). The arsenic content of the human placenta following arsphenamine therapy. Am. J. Obstet. Gynecol..

[36] ATSDR–Toxicological Profile: Arsenic. https://www.atsdr.cdc.gov/toxprofiles/tp.asp?id=22&tid=3.

[37] Bloom M.S., Fitzgerald E.F., Kim K., Neamtiu I., Gurzau E.S. (2010). Spontaneous pregnancy loss in humans and exposure to arsenic in drinking water. Int. J. Hyg. Environ. Health.

[38] Milton A.H., Smith W., Rahman B., Hasan Z., Kulsum U., Dear K., Ali A. (2005). Chronic arsenic exposure and adverse pregnancy outcomes in bangladesh. Epidemiology.

[39] Rahman A., Vahter M., Ekström E.C., Rahman M., Golam Mustafa A.H.M., Wahed M.A., Yunus M., Persson L. (2007). Association of arsenic exposure during pregnancy with fetal loss and infant death: A cohort study in Bangladesh. Am. J. Epidemiol..

[40] Ahmed S.M., Noble B.N., Joya S.A., Ibn Hasan M.O.S., Lin P.I., Rahman M.L., Kile M.L. (2019). A Prospective Cohort Study Examining the Associations of Maternal Arsenic Exposure with Fetal Loss and Neonatal Mortality. Am. J. Epidemiol..

[41] Quansah R., Armah F.A., Essumang D.K., Luginaah I., Clarke E., Marfoh K., Dzodzomenyo M. (2015). Association of Arsenic with Adverse Pregnancy Outcomes/Infant Mortality: A Systematic Review and Meta-Analysis. Environ. Health Perspect..

[42] Hopenhayn-Rich C., Browning S.R., Hertz-Picciotto I., Ferreccio C., Peralta C., Gibb H. (2000). Chronic arsenic exposure and risk of infant mortality in two areas of Chile. Environ. Health Perspect..

[43] von Ehrenstein O.S., Guha Mazumder D.N., Hira-Smith M., Ghosh N., Yuan Y., Windham G., Ghosh A., Haque R., Lahiri S., Kalman D. (2006). Pregnancy outcomes, infant mortality, and arsenic in drinking water in West Bengal, India. Am. J. Epidemiol..

[44] Myers S.L., Lobdell D.T., Liu Z., Xia Y., Ren H., Li Y., Kwok R.K., Mumford J.L., Mendola P. (2010). Maternal drinking water arsenic exposure and perinatal outcomes in Inner Mongolia, China. J. Epidemiol. Community Health.

[45] Milton A.H., Hussain S., Akter S., Rahman M., Mouly T.A., Mitchell K. (2017). A Review of the Effects of Chronic Arsenic Exposure on Adverse Pregnancy Outcomes. Int. J. Environ. Res. Public Health.

[46] Bloom M.S., Neamtiu I.A., Surdu S., Pop C., Lupsa I.R., Anastasiu D., Gurzau E.S. (2014). Consumption of low-moderate level arsenic contaminated water does not increase spontaneous pregnancy loss: A case control study. Environ. Health.

[47] Smith A.H., Lingas E.O., Rahman M. (2000). Contamination of drinking-water by arsenic in Bangladesh: A public health emergency. Bull World Health Organ..

[48] Frisbie S.H., Ortega R., Maynard D.M., Sarkar B. (2002). The concentrations of arsenic and other toxic elements in Bangladesh’s drinking water. Environ. Health Perspect..

[49] Hopenhayn-Rich C., Biggs M.L., Smith A.H. (1998). Lung and kidney cancer mortality associated with arsenic in drinking water in Córdoba, Argentina. Int. J. Epidemiol..

[50] Wasserman G.A., Liu X., LoIacono N.J., Kline J., Factor-Litvak P., van Geen A., Graziano J.H. (2014). A cross-sectional study of well water arsenic and child IQ in Maine schoolchildren. Environ. Health.

[51] Golub M.S., Macintosh M.S., Baumrind N. (1998). Developmental and reproductive toxicity of inorganic arsenic: Animal studies and human concerns. J. Toxicol. Environ. Health Part B.

[52] Kabir T., Anwar S., Taslem Mourosi J., Hossain J., Rabbane M.G., Rahman M.M., Tahsin T., Hasan N., Shill M.C., Hosen M.J. (2020). Arsenic hampered embryonic development: An in vivo study using local Bangladeshi Danio rerio model. Toxicol. Rep..

[53] Walter I., Schwerdtle T., Thuy C., Parsons J.L., Dianov G.L., Hartwig A. (2007). Impact of arsenite and its methylated metabolites on PARP-1 activity, PARP-1 gene expression and poly(ADP-ribosyl)ation in cultured human cells. DNA Repair.

[54] Murphy S.K., Jirtle R.L. (2000). Imprinted genes as potential genetic and epigenetic toxicologic targets. Environ. Health Perspect..

[55] Vega L., Montes de Oca P., Saavedra R., Ostrosky-Wegman P. (2004). Helper T cell subpopulations from women are more susceptible to the toxic effect of sodium arsenite in vitro. Toxicology.

[56] Zhang T.C., Schmitt M.T., Mumford J.L. (2003). Effects of arsenic on telomerase and telomeres in relation to cell proliferation and apoptosis in human keratinocytes and leukemia cells in vitro. Carcinogenesis.

[57] Waalkes M.P., Liu J., Chen H., Xie Y., Achanzar W.E., Zhou Y.S., Cheng M.-L., Diwan B.A. (2004). Estrogen signaling in livers of male mice with hepatocellular carcinoma induced by exposure to arsenic in utero. J. Natl. Cancer Inst..

[58] Bodwell J.E., Kingsley L.A., Hamilton J.W. (2004). Arsenic at very low concentrations alters glucocorticoid receptor (GR)-mediated gene activation but not GR-mediated gene repression: Complex dose-response effects are closely correlated with levels of activated GR and require a functional GR DNA binding domain. Chem. Res. Toxicol..

[59] Chattopadhyay S., Ghosh S., Chaki S., Debnath J., Ghosh D. (1999). Effect of sodium arsenite on plasma levels of gonadotrophins and ovarian steroidogenesis in mature albino rats: Duration-dependent response. J. Toxicol. Sci..

[60] Rassen J.A., Shelat A.A., Myers J., Glynn R.J., Rothman K.J., Schneeweiss S. (2012). One-to-many propensity score matching in cohort studies. Pharmacoepidemiol. Drug Saf..

[61] Stuart E.A., Green K.M. (2008). Using Full Matching to Estimate Causal Effects in Nonexperimental Studies: Examining the Relationship between Adolescent Marijuana Use and Adult Outcomes. Dev. Psychol..

[62] Hansen B.B. (2004). Full Matching in an Observational Study of Coaching for the SAT. J. Am. Stat. Assoc..

[63] Stürmer T., Joshi M., Glynn R.J., Avorn J., Rothman K.J., Schneeweiss S. (2006). A review of the application of propensity score methods yielded increasing use, advantages in specific settings, but not substantially different estimates compared with conventional multivariable methods. J. Clin. Epidemiol..

[64] Desai R.J., Rothman K.J., Bateman B.T., Hernandez-Diaz S., Huybrechts K.F. (2017). A Propensity score based fine stratification approach for confounding adjustment when exposure is infrequent. Epidemiology.

[65] Kile M.L., Houseman E.A., Breton C.V., Quamruzzaman Q., Rahman M., Mahiuddin G., Christiani D.C. (2007). Association between total ingested arsenic and toenail arsenic concentrations. J. Environ. Sci. Health A Tox. Hazard Subst. Environ. Eng..

