# Supplementary material for: Testing the Limit: Evaluating Drinking Water Arsenic Regulatory Levels Based on Adverse Pregnancy Outcomes in Bangladesh
Source: Toxics. 2022 Oct 11;10(10):600. doi: 10.3390/toxics10100600 (PMC9609177; doi:10.3390/toxics10100600)
Supplement: Supplementary file 1 [file toxics-10-00600-s001.zip › toxics-1941715-supplementary.pdf]

Title: Testing the Limit: Evaluating Drinking Water Arsenic Regulatory Levels Based on Adverse Pregnancy Outcomes in Bangladesh

Authors: Faye V. Andrews[1,2], Adam Branscum[1], Perry Hystad[1], Ellen Smit[1], Sakila Afroz[3], Mostofa Golam[3], Omar Sharif [3], Mohammad Rahman[3], Quazi Quamruzzaman[3], David C. Christiani[4], Molly L. Kile[1]

Author affiliations: [1] School of Biological and Population Health Sciences, College of Public Health and Human Sciences, Oregon State University. [2] Oregon Clinical and Translational Research Institute, Oregon Health and Sciences University. [3] Dhaka Community Hospital Trust, Dhaka, Bangladesh. [4] Harvard T.H. Chan School of Public Health, Department of Environmental Health, Harvard University.

Supplement:

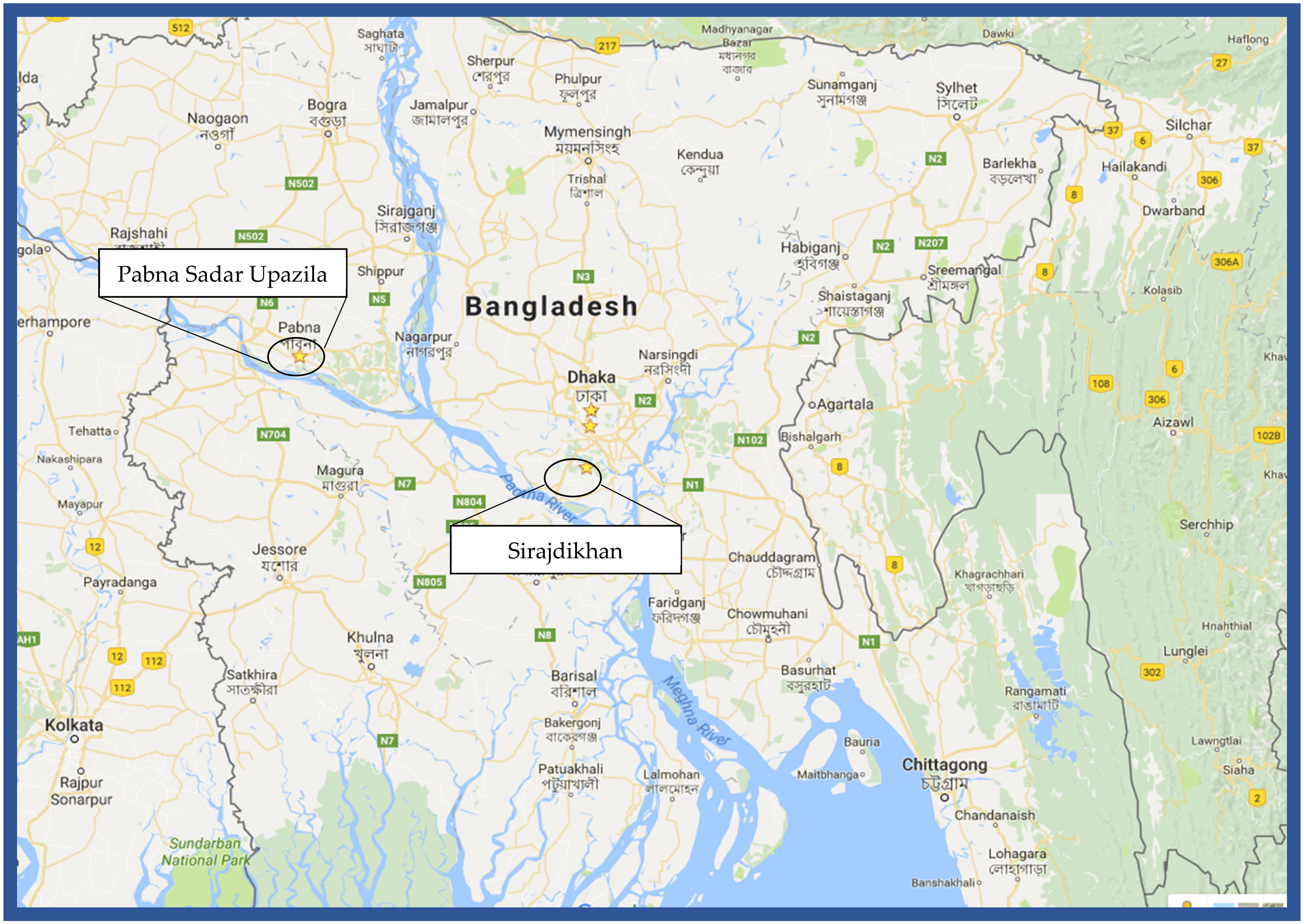

Figure S1. Study locations of Pabna Sadar and Sirajdikhan Upazilas in Bangladesh.

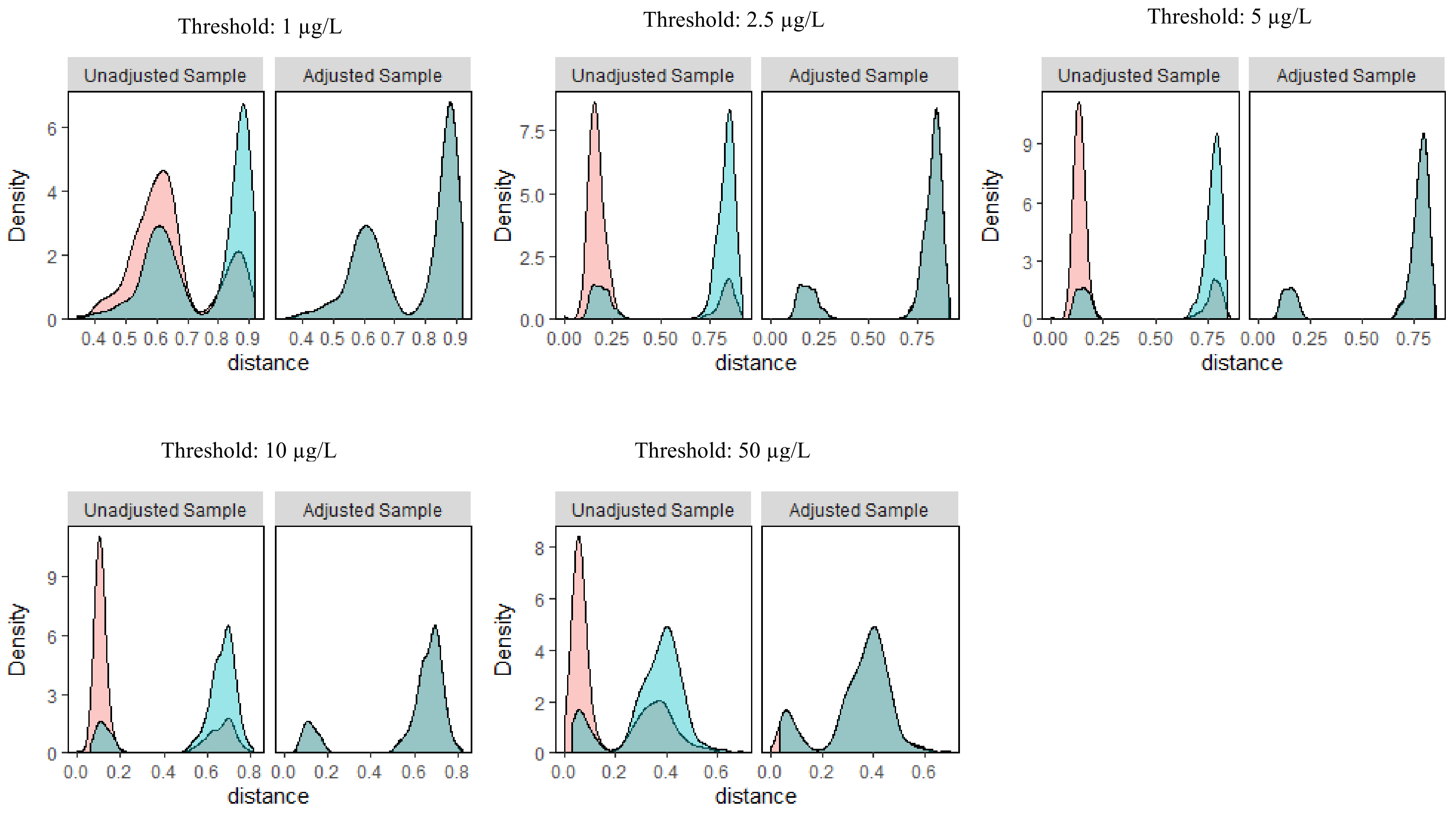

Figure S2. Distributional balance of propensity scores by treatments of above and below thresholds of 1 µg/L, 2.5 µg/L, 5 µg/L, 10 µg/L, and 50 µg/L.

Table S1. Characteristics of Pregnancy Cohort for unmatched and propensity score matched groups for Miscarriage Outcome.

	Unmatched cohort		Matched cohort*	
	**Live Births (N=1130)**	**Miscarriage (N=132)**	**Live Births (N=1120)**	**Miscarriage (N=117)**
**Age (years)**				
Mean (SD)	23.0 (4.22)	23.1 (4.45)	23.0 (4.22)	22.8 (4.06)
Median [Min, Max]	22.0 [18.0, 41.0]	22.0 [18.0, 35.0]	22.0 [18.0, 41.0]	22.0 [18.0, 35.0]
**BMI (Kg/m^2)**				
Mean (SD)	20.5 (3.23)	20.8 (3.18)	20.5 (3.22)	21.0 (3.16)
Median [Min, Max]	20.0 [13.4, 36.0]	20.4 [12.6, 29.0]	20.0 [13.4, 36.0]	20.7 [12.6, 29.0]
**Education**				
No school attendance	159 (14.1%)	24 (18.2%)	159 (14.2%)	18 (15.4%)
Primary School	365 (32.3%)	45 (34.1%)	362 (32.3%)	42 (35.9%)
Secondary School and Higher Education	606 (53.6%)	63 (47.7%)	599 (53.5%)	57 (48.7%)
**Monthly Household Income (Taka)**				
>3,000	188 (16.6%)	11 (8.3%)	188 (16.8%)	11 (9.4%)
3,001-4,000	298 (26.4%)	25 (18.9%)	296 (26.4%)	25 (21.4%)
4,001-5,000	336 (29.7%)	43 (32.6%)	335 (29.9%)	43 (36.8%)
5,001+	301 (26.6%)	38 (28.8%)	301 (26.9%)	38 (32.5%)
Missing	7 (0.6%)	15 (11.4%)	--	--
**Cook Fuel Type**				
Clean Fuel	778 (68.8%)	86 (65.2%)	772 (68.9%)	77 (65.8%)
Less Clean Fuel	346 (30.6%)	46 (34.8%)	345 (30.8%)	40 (34.2%)
Refused	3 (0.3%)	0 (0%)	3 (0.3%)	0 (0%)
Missing	3 (0.3%)	0 (0%)	--	--
**Risk of subsequent miscarriage/stillbirth**				
First Born or No Previous	922 (81.6%)	105 (79.5%)	914 (81.6%)	95 (81.2%)

	Unmatched cohort		Matched cohort*	
	**Live Births (N=1130)**	**Miscarriage (N=132)**	**Live Births (N=1120)**	**Miscarriage (N=117)**
Previous Miscarriage/Stillbirth	208 (18.4%)	27 (20.5%)	206 (18.4%)	22 (18.8%)
**Number of previous births**				
First Born	536 (47.4%)	68 (51.5%)	531 (47.4%)	62 (53.0%)
2nd or Greater Born	594 (52.6%)	64 (48.5%)	589 (52.6%)	55 (47.0%)
**Clinic Location**				
Pabna	555 (49.1%)	48 (36.4%)	553 (49.4%)	33 (28.2%)
Sirajdikhan	575 (50.9%)	84 (63.6%)	567 (50.6%)	84 (71.8%)
**Drinking Water Manganese (µg/L)**				
Mean (SD)	722 (704)	708 (715)	714 (692)	721 (736)
Median [Min, Max]	590 [0.500, 4720]	620 [1.00, 3500]	590 [0.500, 4720]	639 [1.00, 3500]
**Drinking Water Arsenic cut off 1 µg/L**				
Low	312 (27.6%)	36 (27.3%)	309 (27.6%)	35 (29.9%)
High	818 (72.4%)	96 (72.7%)	811 (72.4%)	82 (70.1%)
**Drinking Water Arsenic cut off 2.5 µg/L**				
Low	576 (51.0%)	73 (55.3%)	569 (50.8%)	70 (59.8%)
High	554 (49.0%)	59 (44.7%)	551 (49.2%)	47 (40.2%)
**Drinking Water Arsenic cut off 5 µg/L**				
< 5 ug/L	624 (55.2%)	78 (59.1%)	617 (55.1%)	74 (63.2%)
≥ 5 ug/L	506 (44.8%)	54 (40.9%)	503 (44.9%)	43 (36.8%)
**Drinking Water Arsenic cut off 10 µg/L**				
< 10 ug/L	699 (61.9%)	88 (66.7%)	691 (61.7%)	83 (70.9%)
≥ 10 ug/L	431 (38.1%)	44 (33.3%)	429 (38.3%)	34 (29.1%)
**Drinking Water Arsenic cut off 50 µg/L**				
< 50 ug/L	886 (78.4%)	108 (81.8%)	878 (78.4%)	99 (84.6%)
≥ 50 ug/L	244 (21.6%)	24 (18.2%)	242 (21.6%)	18 (15.4%)

*Matched using full matching in propensity scores

Table S2. Sensitivity analysis comparing traditional modeling approaches and propensity score matching for miscarriage outcome.

Type of Model

Arsenic cut off level	***Unadjusted Logistic Regression***		****Adjusted Logistic Regression***		****Propensity score full matching***		****Propensity score nearest neighbor matching***	
	Below	***Above***	***Below***	***Above***	***Below***	***Above***	***Below***	***Above***
**1 µg/L**	1	0.89 (0.59 – 1.36)	1	1.24 (0.80 – 1.93)	1	1.10 (0.62 – 1.94)	1	2.63 (0.56 – 12.39)
**2.5 µg/L**	1	0.69 (0.47 – 1.03)	1	1.48 (0.88 – 2.49)	1	**1.90 (1.07 – 3.38)**	1	1.47 (0.90 – 2.39)
**5 µg/L**	1	0.71 (0.48 – 1.06)	1	1.53 (0.90 – 2.60)	1	**2.73 (1.49 – 5.02)**	1	1.58 (0.96 – 2.60)
**10 µg/L**	1	0.66 (0.43 – 1.01)	1	1.23 (0.72 – 2.10)	1	1.18 (0.60 – 2.30)	1	1.21 (0.73 – 2.01)
**50 µg/L**	1	0.66 (0.39 – 1.12)	1	1.05 (0.58 – 1.89)	1	0.66 (0.33 – 1.33)	1	1.01 (0.50 – 2.05)

*Adjusted for age, BMI, income, education, household cook fuel type, location of clinic, risk of previous miscarriage or stillbirth if not first child, parity, and drinking water manganese.

Table S3. Propensity Score Standard Mean Differences between pre-matching and post-matching with full and nearest neighbor methods for arsenic cut-off values of 2.5 and 5 μg/L.

	Arsenic Cut-off Value: 2.5 μg/L			Arsenic Cut-off Value: 5 μg/L		
**Covariate**	*Pre-matching*	*Post-matching Full*	*Post-matching Nearest neighbor*	*Pre-matching*	*Post-matching Full*	*Post-matching Nearest neighbor*
Distance	1.7694	-0.0008	1.7231	1.7929	0.0001	1.6208
Location – Pabna	1.7104	-0.0000	1.6833	1.7606	0.0000	1.6264
Location – Sirajdikhan	-1.7104	-0.0000	-1.6833	-1.7606	0.0000	-1.6264
BMI (Body Mass Index)	-0.3285	-0.1386	-0.2118	-0.3006	-0.1717	-0.0630
Age of participant	0.0250	-0.2070	0.0408	-0.0061	-0.2234	0.0617
Education – No school attendance	0.2041	-0.2071	0.1942	0.1931	-0.0603	0.1509
Education – Primary School attendance	-0.2739	0.0235	-0.2351	-0.2634	0.0376	-0.1541
Education – Secondary School and Higher Education	0.0838	0.1405	0.0572	0.0821	0.0138	0.0184
Income - >3,000 Taka	0.3885	-0.0501	0.3759	0.3718	-0.1169	0.3168
Income - 3,001- 4,000 Taka	0.2355	0.0448	0.2086	0.2072	0.0283	0.0948
Income – 4,001 – 5,000 Taka	-0.2206	-0.0138	-0.2299	-0.1700	-0.0056	-0.2494
Income – 5,000 + Taka	-0.4703	0.0176	-0.4130	-0.4831	0.1059	-0.1892
Clean Cook Fuel	-0.2437	0.0744	-0.2349	-0.2741	-0.0626	-0.1691
Less Clean Cook Fuel	0.2534	-0.0734	0.2349	0.2830	0.0631	0.1691
Cook fuel – refused to answer	-0.0954	-0.0093	0.0000	-0.0883	-0.0056	0.0000
Drinking water manganese	0.0620	-0.2994	0.0405	0.0464	-0.2133	-0.0230
First Born or No Previous Miscarriage/Stillbirth	-0.1700	-0.0009	-0.1532	-0.1231	0.0019	-0.1075
Previous Miscarriage/Stillbirth	0.1700	0.0009	0.1532	0.1231	-0.0019	0.1075
Parity – first born	-0.1808	0.1628	-0.1991	-0.1771	0.0021	-0.2071
Parity – 2nd or greater born	0.1808	-0.1628	0.1991	0.1771	-0.0021	0.2071
